# Tranilast Can be a Useful Addition to the Limited Anti-Epidermolysis Bullosa Weaponry

**DOI:** 10.15171/apb.2017.001

**Published:** 2017-04-13

**Authors:** Mohammad Mahdi Parvizi, Mohammad Reza Namazi

**Affiliations:** ^1^Molecular Dermatology Research Center, Shiraz University of Medical Sciences, Shiraz, Iran.; ^2^Essence of Parsiyan Wisdom Institute, Phytopharmaceutical and Traditional Medicine Incubator, Shiraz University of Medical Sciences, Shiraz, Iran.

## Dear editor

Epidermolysis bullosa (EB) is a hereditary genetic disease characterized by varying degrees of skin and mucosa fragility.^1^ The cause of this disease is mutation in skin structural proteins. There are four major types of EB, including EB simplex (EBS), junctional EB (JEB), dystrophic EB (DEB) and Kindler’s Syndrome based on ultrastructural mutation level in tissue of skin and mucosa.^2^ Patients with EB suffer from many complications of this disease such as infection, deformities of upper and lower extremities, gastrointestinal stricture, dysphasia and odynophagia due to narrowing of the esophagus, widespread skin ulceration that makes them susceptible to skin cancer, urinary tract dysfunction and kidney fibrosis. Itch is another main problem of these patients that can disrupt their normal function.^3^ Fibrosis of both small and large intestines of these patients can cause malabsorbtion, constipation, failure to thrive and weakness. Fibrosis due to severe skin ulcers and inflammation leads to joint contractures in EB patient and makes them defective and decreases the quality of life in these patients.^4,5^

Recent in-vivo and in-vitro studies confirm the pivotal role TGF-β in fibroblast proliferation and collagen synthesis leading to fibrosis.^6^ Suppressing the TGF-β via reducing fibrosis can reduce the problems of patents with EB.^7^

Several studies showed that tranilast, N-(3,4-demethoxycinnamyl)-anthranilic acid, is a mast-cell stabilizing antihistamine and anti-inflammatory and anti-oxidant drug capable of suppressing the TGF-β.^8,9^ Because of these effects, this drug is used for the treatment of atopic dermatitis and scleroderma.^10^ Anti-fibrotic effect of tranilast is shown in myocardial fibrosis in mice with viral myocarditis.^11^ Anti-cancerogenic effect of this medicine is also determined in-vivo.^12^

Therefore, we conclude that administering this safe drug for patients with EB, specially in DEB and JEB, can mitigate the fibrotic complications of this disease, such as fibrosis of the skin, kidney, esophagus and bowel as well as alleviating the pruritus and preventing the cutaneous cancers. It introduces a novel and very safe medication for a very debilitating disease and can encourage researchers, especially those who have access to this drug, to conduct further trials on this topic.

## Ethical Issues

Not applicable.

## Conflict of Interest

The authors declare that there is no conflict of interests regarding the publication of this paper.
